# The Opposites Attract Study of viral load, HIV treatment and HIV transmission in serodiscordant homosexual male couples: design and methods

**DOI:** 10.1186/1471-2458-14-917

**Published:** 2014-09-04

**Authors:** Benjamin R Bavinton, Fengyi Jin, Garrett Prestage, Iryna Zablotska, Kersten K Koelsch, Nittaya Phanuphak, Beatriz Grinsztejn, David A Cooper, Christopher Fairley, Anthony Kelleher, Kathy Triffitt, Andrew E Grulich, the Opposites Attract Study Group

**Affiliations:** The Kirby Institute, University of New South Wales, 2052 Sydney, NSW Australia; Australian Research Centre in Sex, Health and Society, La Trobe University, 3000 Melbourne, Victoria Australia; St. Vincent’s Hospital, Sydney, 2010 Darlinghurst, NSW Australia; Thai Red Cross AIDS Research Centre, 10330 Phathumwan, Bangkok, Thailand; Instituto de Pesquisa Clínica Evandro Chagas, Manguinhos, 21040-360 Rio de Janeiro, Brazil; Melbourne Sexual Health Centre, 3053 Carlton, Victoria Australia; Monash University, 3800 Clayton, Victoria Australia; St. Vincent’s Centre for Applied Medical Research, St. Vincent’s Hospital, 2010 Darlinghurst, NSW Australia

**Keywords:** HIV treatment as prevention, Serodiscordant, Homosexual, HIV transmission, Antiretroviral therapy

## Abstract

**Background:**

Studies in heterosexual HIV serodiscordant couples have provided critical evidence on the role of HIV treatments and undetectable viral load in reducing the risk of HIV transmission. There is very limited data on the risk of transmission from anal sex in homosexual male serodiscordant couples.

**Methods/Design:**

The *Opposites Attract Study* is an observational prospective longitudinal cohort study of male homosexual serodiscordant partnerships running from 2012 to 2015 and conducted in clinics throughout Australia, Brazil and Thailand. Couples attend two or more clinic visits per year. The HIV-positive partner’s viral load is tested and the HIV-negative partner is tested for HIV antibodies at every clinic visit. Results from any tests for sexually transmitted infections are also collected. Detailed behavioural questionnaires are completed by both partners at the time of each visit. The primary research question is whether HIV incidence is lower in those couples where the HIV-positive partner is receiving HIV treatment compared to couples where he is not receiving treatment. A voluntary semen sub-study will examine semen plasma viral load in a subsample of HIV-positive partners in Sydney, Rio de Janeiro and Bangkok. In cases of seroconversion of the initially HIV-negative partner, phylogenetic analysis will be conducted at the end of the study on virus from stored blood samples from both partners to determine if the infection came from the HIV-positive study partner. Men in new serodiscordant relationships will specifically be targeted for recruitment.

**Discussion:**

This study will provide critical data on the reduction in HIV transmission risk associated with being on HIV treatment in homosexual male serodiscordant couples in different regions of the world. Data from men in new relationships will be particularly valuable given that the highest transmission risk is in the first year of serodiscordant relationships. Furthermore, the detailed behavioural and attitudinal data from the participant questionnaires will allow exploration of many contextual factors associated with HIV risk, condom use and the negotiation of sexual practice within couples.

## Background

Studies in heterosexual HIV serodiscordant couples have provided critical evidence on the role of HIV treatments and undetectable viral load in reducing the risk of HIV transmission. A number of observational studies, primarily in African heterosexual serodiscordant couples, have demonstrated a link between HIV treatment, undetectable viral load and reduced transmission risk [1]. In 2011, the early results of a randomised clinical trial, HPTN 052, provided conclusive evidence that when the HIV-positive partner is on antiretroviral therapy (ART), transmission risk is dramatically reduced by 96% in heterosexual serodiscordant couples [2].

The strategy of reducing HIV transmission risk by placing the HIV-positive partner on ART irrespective of stage of HIV infection has widely become known as ‘treatment as prevention’ (TasP) [3–5]. Despite several studies demonstrating the effectiveness of TasP in heterosexual serodiscordant couples, there is currently no conclusive evidence on the relationship between ART and transmission in male homosexual serodiscordant couples [6, 7]. Given that the risk of HIV transmission via anal intercourse is about 10 times greater than for vaginal intercourse [8], it cannot be assumed that the effectiveness of TasP will be identical for the two types of sex. As anal intercourse is not uncommon among heterosexuals (for example, 20.9% of adult heterosexual men and 15.1% of adult heterosexual women in Australia report anal sex in their lifetime [9]), findings in homosexual men may also have broader relevance for the effectiveness of TasP in heterosexuals.

Internationally, only one study has reported findings on TasP in homosexual male serodiscordant couples: the *Partners of people on ART – a New Evaluation of the Risks (PARTNER) Study* in the United Kingdom and Europe [7]. The interim analysis presented in early 2014 provided promising but not yet conclusive results. Amongst 308 homosexual male serodiscordant couples, no phylogenetically linked HIV transmissions were observed in two years of follow-up. However, the results were statistically consistent with a possible risk of up to 1.17% per year for couples who reported any unprotected anal intercourse (UAI), and up to 1.97% per year in those couples where receptive UAI (with or without ejaculation) was reported [10].

### Research questions

The *Opposites Attract Study* addresses the following questions, in serodiscordant male homosexual partnerships:

1. Is HIV incidence lower in those couples where the HIV-positive partner is receiving ART, compared with not receiving ART?
2. Is HIV incidence higher in those couples where the HIV-positive partner has detectable blood plasma viral load (BPVL) compared to those couples where he has undetectable BPVL?
3. Is BPVL used in negotiating unprotected anal intercourse within couples?
4. Is HIV transmission possible when the HIV-positive partner has undetectable BPVL but either partner has a relevant sexually transmitted infection (STI)?
5. In a subsample of couples, is HIV incidence related to semen plasma viral load (SPVL) in the HIV-positive partner?
6. In a subsample of HIV-positive men, is BPVL associated with SPVL?

## Methods/Design

### Summary of study design

*Opposites Attract* is an observational prospective longitudinal cohort study of male homosexual serodiscordant partnerships, coordinated by the Kirby Institute at the University of New South Wales, Australia. The study is conducted through several high HIV caseload clinics throughout Australia and in Bangkok, Thailand and Rio de Janeiro, Brazil. The study involves attendance at two or more clinic visits per year followed by completion of online computer-assisted self-interviews after each visit. At clinic visits, data are collected on HIV BPVL and CD4 T-cell count in the HIV-positive partner, HIV antibodies in the HIV-negative partner, and STI test results in both partners. Samples of blood for storage of plasma, serum and buffy coat are taken from both partners at baseline, once a year during follow-up (from the HIV-positive partner only), and at seroconversion of the initially HIV-negative partner should that occur. Anal swabs are taken for storage from the HIV-negative partner at baseline and if seroconversion occurs. In a voluntary sub-study at sites in Sydney, Bangkok and Rio de Janeiro, HIV-positive partners attending clinics are invited to provide a semen sample at the time of each clinic visit. Enrolment commenced in April 2012 and under current funding arrangements, follow-up will continue until the end of 2015.

### Sample size

The sample size calculations were based on the hypotheses relating to BPVL and HIV incidence. The calculations were performed using data on HIV incidence in HIV-negative men with HIV-positive primary regular partners from previous Kirby Institute studies [11, 12]. It was not possible to be as precise in estimating the study’s power to test hypotheses concerning SPVL. Two types of power calculation were performed, to consider situations in which either zero or at least one linked seroconversion occur in the study. This was because the study may or may not identify cases of linked HIV transmissions within the serodiscordant couples.

1. *At least 1 phylogenetically linked HIV seroconversion is identified in the cohort:*

We examined various plausible scenarios where the study would have at least 80% power to detect at least a 75% reduction in the incidence of HIV cases in those whose partners have undetectable as compared to detectable BPVL. For the primary power calculation, based on Australian research we assumed that 60% of HIV-positive partners will have undetectable BPVL [13]. We also assumed that the HIV incidence in HIV-negative men whose partners have detectable BPVL will be 5.0 per 100 person-years. This assumption was derived from the incidence observed in the *Health In Men (HIM)* study, our previous cohort study of HIV-negative gay men in Sydney conducted between 2001 and 2007 [12]. The incidence of HIV in negative men whose partners had detectable viral load could not be directly calculated in the *HIM* study due to lack of completeness of data on the BPVL of HIV-positive partners. However, we estimated an incidence of around 5 per 100 person-years in partnerships where the HIV-positive partner had detectable BPVL. This was based on two factors: the HIV incidence of 2.2 per 100 person-years in men with HIV-positive primary regular partners in *HIM* [14], and the fact that in 60% of these relationships, the BPVL would have been undetectable and therefore have lower HIV incidence than 2.2 per 100 person-years. In this scenario, we estimated 18 incident HIV infections would be required, requiring a total of 631 couple-years of follow-up to enable 80% statistical power to detect at least a 75% reduction in HIV incidence in men whose partners have undetectable BPVL.

For other scenarios we assumed greater proportions of HIV-positive partners with undetectable viral load. This was based on the possibility that more HIV-positive men may commence treatment following the publication of results from TasP studies in heterosexuals. These scenarios and the associated sample sizes are presented in Table 1. In summary, a sample size of 640 couple-years of follow-up will result in a statistical power of approximately 80% even if the proportion of HIV-positive partners with undetectable BPVL increases to 70% or 80%. However, if this proportion increases to 90% of HIV-positive men, the study will be powered to detect an 80% reduction in HIV incidence in negative men whose partners have undetectable BPVL, which means the HIV incidence in these men will be 1.0 per 100 person-years or lower.

2. *No phylogenetically linked HIV seroconversions are identified in the cohort (transmission rate = 0 per 100 person-years):*

As mentioned, recent research findings relating to the effectiveness of TasP [2, 10] may lead to a greater percentage of HIV-positive gay men in serodiscordant relationships electing to receive ART and thus having undetectable BPVL. For the purposes of this calculation, we have assumed that this may increase to 80% or 90% of all serodiscordant couples. If this is the case, and if effective antiretroviral treatment is as successful at reducing the risk of HIV transmission in gay men as was found among heterosexuals in HPTN 052 (i.e. 96% reduction), we may find no phylogenetically linked seroconversions in this study. In this case, the analysis will focus on calculating the upper limit of the confidence interval of an HIV incidence of 0 per 100 person years in couples where the HIV-positive partner has undetectable BPVL.

Assuming that the study achieves 640 couple-years of follow-up, and no phylogenetically related transmissions are documented, the upper limit of the 97.5% single-sided HIV incidence in partnerships where the HIV-positive partner has undetectable BPVL would be 0.72 and 0.64 per 100 person-years, if the proportion of HIV-positive participants with undetectable BPVL is 80% and 90% respectively.

**Table 1:** **Serodiscordant couples sample size calculations**

Percentage of HIV men who are on ART and have undetectable BPVL	Reduction in HIV incidence in HIV-negative men with partners with undetectable BPVL	Required number of HIV seroconversion events	Total sample size required
**60%**	0.75	18	631
	0.85	11	436
	0.95	7	305
**70%**	0.75	15	624
	0.85	9	413
	0.95	5	272
**80%**	0.75	14	692
	0.85	7	429
	0.95	4	256
**90%**	0.75	17	1008
	0.85	7	563
	0.95	3	276

### Eligibility

Homosexual men in sexual partnerships meeting the following criteria are eligible to enroll in the study: (1) Both partners aged 18 years or over; (2) One partner is HIV-positive and the other partner HIV-negative at baseline; (3) Partners must have anal sex with each other at least once per month on average; (4) Partners must believe they will still be having anal sex with each other by the time of the HIV-positive partner’s next viral load test (that is, between three to six months after baseline); and (5) Both partners must agree to attend clinic visits at least twice per year. HIV-positive partners can enroll in the study irrespective of whether they are taking ART or not, or whether their BPVL is undetectable or detectable. Couples can enroll irrespective of whether they engage in unprotected or protected anal intercourse with each other.

### Clinical sites

As of September 2014, fourteen high HIV caseload public (n=6) and private (n=8) clinics in Australia are participating in the study, along with one clinic in Rio de Janeiro, Brazil, and another clinic in Bangkok, Thailand. New sites may be initiated at a later time.

### Recruitment

Clinics are free to employ a range of recruitment strategies to identify and recruit potential participants, depending on what is deemed appropriate for each clinic. Mostly, couples are recruited when clinicians raise the study directly with HIV-positive patients or HIV-negative patients known to have an HIV-positive regular partner. Interested patients are given information about the study and are asked to speak to their regular partner about participation. They are contacted by the clinic-based study coordinator and an enrolment visit is arranged if the couple is both interested and eligible. Community-based study promotion is also implemented in the form of print and online advertising, attendance and/or presentations at community events, and engagement with community-based partner organisations.

### Enrolment and clinic visits

In Australia, couples are enrolled at the clinic the HIV-positive partner attends for routine clinical management. In Brazil and Thailand, couples are enrolled at the study clinic. The study is clearly explained, and consent forms signed. Participants must sign the consent forms while their partner is outside the room, to minimise the risk of coerced participation. Both partners complete an HIV transmission knowledge quiz and education process to ensure they understand how HIV is transmitted in the context of serodiscordant male-to-male sex. They are encouraged to read the *Opposites Attract* HIV transmission factsheet before attending the enrolment visit.

Once enrolled, both partners undergo a baseline clinic visit, involving pathology samples testing (viral load, CD4, HIV antibodies, STIs) and the collection of biological samples for storage. Follow-up visits occur each time regular viral load monitoring of the HIV-positive partner occurs, a minimum of two times per year. Partners do not need to attend their clinic visits at the same time. However, they must attend within two weeks of each other.

Table 2 indicates all procedures associated with each clinic visit. A viral load test is required for an HIV-positive partner’s visit to be considered a study visit, while an HIV antibody test is required for an HIV-negative partner’s visit to be considered a study visit. In Australia, STI tests are conducted as indicated by Australian guidelines [15]. In Brazil and Thailand, syphilis, gonorrhoea and chlamydia tests are conducted at every study visit. At certain visits, extra blood samples are taken for storage from both partners, and an extra anal swab taken from HIV-negative partners. These samples are processed and stored at local laboratories and shipped to the central laboratory in batches. The blood samples allow for later phylogenetic testing of the partners’ virus in cases of seroconversion of the initially HIV-negative partner. In addition, the specimens may potentially be used to identify genetic factors associated with resistance to HIV acquisition in HIV-negative partners. The anal swabs are taken to later test for rectal herpes simplex virus (HSV) and human papillomavirus (HPV). In Sydney, Rio de Janeiro and Bangkok, HIV-positive participants can elect to join the Semen Sub-Study whereby they provide a semen sample at the time of each clinic visit. The semen samples are processed and stored for later viral load and phylogenetic testing.

**Table 2:** **Procedures associated with each clinic visit**

	Baseline visit	First visit of calendar year	All other follow-up visits	Seroconversion visit ^1^
**HIV-Positive partner**				
Viral load test	X	X	X	X
CD4 Count	X	X	X	X
STI tests^3^	X	X	X	X
Blood samples for storage	X	X		X
Semen sample *(optional)* ^2^	X	X	X	X
**HIV-Negative partner**				
HIV antibody test	X	X	X	X
STI tests^3^	X	X	X	X
Blood samples for storage	X			X
Anal swab for storage	X			X

^1^Seroconversion visits only occur if the HIV-negative partner seroconverts during follow-up.

^2^The semen sub-study is only available at certain sites.

^3^At Australian clinical sites, STI tests are conducted as indicated. In Brazil and Thailand, they are conducted at every visit.

### Data collection

Data are collected via electronic case record forms (eCRFs) completed by clinic staff and online computer-assisted self-interview from both HIV-positive partners and HIV-negative partners. The eCRFs include: enrolment information, clinic visit information (including the HIV-positive partner’s ART regimen), and pathology results information. For HIV-positive partners participating in the Semen Sub-Study, further information is entered upon receipt of the sample. Participants are asked to complete their questionnaires at the clinic visit, or within 48 hours of the visit. As the questionnaires are online, they can be accessed by participants in their homes. Clinicians and clinic staff do not have access to the behavioural questionnaire information regarding their patients, and participants are explicitly informed of this. Table 3 indicates the content categories of the HIV-positive partner and HIV-negative partner questionnaires. Baseline and follow-up questionnaires are very similar. Finally, all initially HIV-negative partners who acquire HIV during follow-up will be invited to participate in a qualitative interview regarding their seroconversion. Further qualitative interviews with other study participants may also be conducted as part of a sub-study.

**Table 3:** **Questionnaire content for HIV-positive and HIV-negative partners**

HIV-Positive partner	HIV-Negative partner
Demographics	Demographics
HIV diagnosis and treatment	HIV testing
ART adherence	STI testing and symptoms
STI testing and symptoms	Relationships and communication
Relationships and communication	Relationship agreements
Attitudes	Partner viral load
Study involvement and motivations	Sexual behaviour with study partner
	Sexual behaviour with other partners
	Group sex
	PEP and PrEP use and knowledge
	Attitudes
	Reasons for using/not using condoms
	Study involvement and motivations

### HIV seroconversion of initially HIV-negative partners

In cases of HIV seroconversion among initially HIV-negative participants, each clinic will follow its standard approach, typically including immediate referral to an HIV specialist for further testing and monitoring, referral to professional and peer-based support services, and discussion of treatment options. The study protocol does not mandate any specific approach to how clinics should deal with HIV seroconversion.

At the end of the study, phylogenetic analysis will be conducted on paired stored blood samples from both partners. Epidemiological relationships will be established by analyses of *env* and *gag-pol* sequences derived from plasma as well as PBMC from these blood samples. Sequence alignment will be performed by using either Clustal X or MUSCLE software. Phylogenetic analyses including patient derived sequences and background sequences derived from the Los Alamos database will assess linkage between different viruses by inferred maximum likelihood phylogeny, neighbour joining methods, pairwise distances, Bayesian inference and bootstrap analyses [16]. Tree visualisation will be performed using, for example, TreeView or UGENE software.

### Data analysis

The primary analyses aim to determine if HIV incidence is reduced in the HIV-negative partner when the HIV-positive partner: (1) is taking ART compared to not taking ART, and (2) has undetectable viral load compared to detectable viral load. These incidence rates will be calculated by dividing the number of phylogenetically linked seroconversions for each group by the total number of person-years of follow-up for that group. If there are no phylogenetically linked seroconversions, the upper limit of the confidence interval around a transmission rate of zero will be determined. Given that laboratories utilise different assays for viral load testing which have various lower limits of detection (e.g. 20, 40, 50, or 150 copies per mL), the precise definition of ‘undetectable’ and ‘detectable’ BPVL differs across sites. This will be taken into account in analysis, and a standardised cut-off may be used for directly measured BPVL (e.g. less than 400 copies per mL).

Two of the study questions relate to SPVL. First, in the subsample of couples included in the Semen Sub-Study, HIV incidence will be compared between couples with detectable versus undetectable SPVL in the HIV-positive partner. Second, within the HIV-positive partners participating in the Semen Sub-Study, BPVL and SPVL will be compared at each study visit. HIV RNA levels in seminal plasma will be determined using an adaptation of an in-house assay for separation of seminal plasma from human samples and the COBAS® AmpliPrep/COBAS® TaqMan® system.

The behavioural and attitudinal data collected from the participant questionnaires will allow for both cross-sectional and longitudinal analyses. A key focus will be on the question of whether gay men in serodiscordant relationships use condoms less often when the HIV-positive partner has undetectable viral load [17]. This will be explored both through directly measured and perceived viral load, both categorically in terms of those who have/do not have UAI as well as by the number and type (insertive/receptive and with/without ejaculation) of acts of UAI, and in relation to attitudes regarding TasP, communication within relationships, and relationship agreements about viral load and sexual practice.

The study data will also allow examination of: the per-contact risk of HIV infection by acts of UAI; concordance of perceived and actual viral load results; impacts of adherence on viral load; use of pre-exposure prophylaxis and post-exposure prophylaxis in HIV-negative partners; comparison of attitudes within couples; the effect of STIs on behaviour and HIV risk; and characteristics of ART use in HIV-positive partners (i.e. starting ART, changing regimens, side effects).

### Ethical committee review

*Opposites Attract* is conducted according to the Australian National Statement on Ethical Conduct in Human Research [18] and the World Medical Association Declaration of Helsinki [19]. It has been approved by several human research ethics committees (HRECs), including at St Vincent’s Hospital (Sydney), The Alfred Hospital (Melbourne), the Prince Charles Hospital (Brisbane), the University of New South Wales (Sydney), the Comitê de Ética em Pesquisa do Instituto de Pesquisa Clinica Evandro Chagas (Rio de Janeiro), and Chulalongkorn University’s Faculty of Medicine (Bangkok). In Australia, the study was also approved by hospital research governance offices before initiation in the public clinics. Further, the study was approved by two ethical review committees based at HIV community-based organisations. Further approvals will be sought if new sites are initiated.

### HIV transmission and legal prosecution

In many countries, HIV transmission is criminalised via public health and/or criminal legislation. The study team sought detailed written legal advice pertaining to each Australian state in which the study is being conducted. Advice was also sought from the international sites regarding local legislation. Several legal protections for HIV-positive partners were implemented. First, all HIV-negative partners are required to sign a declaration stating that they are aware of their sexual partner’s HIV-positive serostatus. Second, at study enrolment, all participants must undertake a quiz on HIV transmission to ensure a high level of knowledge regarding HIV risk in relation to condom use, sexual positioning, STIs, circumcision, withdrawal prior to ejaculation, oral sex, and awareness of post-exposure prophylaxis. If any questions are answered incorrectly, study staff members provide detailed HIV transmission education, and this is documented. Third, at baseline, participants consent to not receiving the results of phylogenetic analysis that would indicate whether or not the new infection was a linked transmission. These results will not be provided to participants or their clinicians under *any* circumstances. Fourth, questionnaires do not ask HIV-positive partners to report sexual risk behaviour; this is asked of the HIV-negative partner only.

## Discussion

While there is strong evidence for the effectiveness of ART to substantially reduce the risk of HIV transmission in heterosexual serodiscordant couples, there is very limited published evidence on TasP within homosexual male serodiscordant couples or in relation to anal intercourse. *Opposites Attract* and the *PARTNER Study* [7] appear to be the only two clinical studies exploring these questions. The *PARTNER Study* focuses on measuring the HIV incidence in both heterosexual and homosexual serodiscordant couples having unprotected anal and/or vaginal intercourse, and is restricted to couples where the HIV-positive partner is receiving ART at baseline. By contrast, *Opposites Attract* will allow the calculation of HIV incidence in couples where HIV-positive partners are on ART or not on ART, and with undetectable versus detectable BPVL (and SPVL in a subsample of couples). Furthermore, the detailed behavioural and attitudinal data from the participant questionnaires will allow exploration of many contextual factors associated with HIV risk, condom use and the negotiation of sexual practice within couples.

Measuring the effect of ART on transmission is impacted by whether serodiscordant couples have unprotected intercourse with each other. In *Opposites Attract*, the decision was made not to restrict enrolment to those couples reporting UAI with each other in the months before baseline (as in the *PARTNER Study*). This was because in a pilot study we conducted in Melbourne, it was found that participants were wary of enrolling or expressing interest in the study if they were required to report having UAI with each other to their clinician. Thus, the eligibility criterion for the main study was changed to the couples having anal intercourse (protected or unprotected) with each other at least once per month on average. The level of risk within the cohort will be monitored over time and changes may be made to this criterion in the future if necessary.

Furthermore, in preparation for *Opposites Attract*, we analysed cohort data of HIV-negative men in Sydney and found that amongst HIV-negative who reported being in a serodiscordant relationship of one year or less, the HIV incidence was approximately 6 per 100 person-years as compared to much lower incidence in those who reported longer relationships, suggesting that the riskiest period for HIV-negative men in serodiscordant relationships is the first year of the relationship [14, 20]. This may be due to the relatively higher frequency of sex in newer relationships [21, 22]. Additionally, partners who stay negative after longer periods of time in a serodiscordant relationship may have reduced susceptibility of some kind, such as having a protective genetic predisposition or acquired immunity through repeated exposure [23]. In any case, this finding suggests that in *Opposites Attract*, men in new relationships need to be targeted to ensure that the effect of ART on transmission can be measured. Restricting participation to ‘stable’ couples that have been together for a specified period of time may have the effect of underestimating the true HIV incidence as the studies may recruit couples once the riskiest period has already passed. Additionally, this may suggest that studies would benefit from targeting non-romantic yet regular sexual relationships (e.g. colloquially known as ‘fuckbuddy’ relationships [24]) as many ‘committed’ homosexual male relationships may begin in this way [25].

## Abbreviations

ART: Antiretroviral therapy
BPVL: Blood plasma viral load
eCRF: Electronic case record form
HIM: Health in men study
HIV: Human immunodeficiency virus
HPTN: HIV prevention trials network
HPV: Human papillomavirus
HREC: Human research ethics committee
HSV: Herpes simplex virus
NSW: New South Wales
PARTNER Study: Partners of people on ART – a New Evaluation of the Risks
PBMC: Peripheral blood mononuclear cell
SPVL: Semen plasma viral load
PEP: Post-exposure prophylaxis
PrEP: Pre-exposure prophylaxis
STI: Sexually transmitted infection
TasP: Treatment as prevention
UAI: Unprotected anal intercourse.
